# Textbook outcome and nomogram-guided approaches for enhancing surgical success in elderly HCC patients: Deciphering the influence of sarcopenia

**DOI:** 10.1007/s13304-024-01992-3

**Published:** 2024-10-07

**Authors:** Jacopo Lanari, Amalia Lupi, Ilaria Billato, Remo Alessandris, Filippo Crimì, Silvia Caregari, Alessia Pepe, Francesco Enrico D’Amico, Alessandro Vitale, Emilio Quaia, Umberto Cillo, Enrico Gringeri

**Affiliations:** 1 Department of Surgical, Oncological and Gastroenterological Sciences, University of Padua, Padua, Italy; 2 General Surgery 2, Hepato-Pancreato-Biliary Surgery and Liver Transplantation, Padua University Hospital, Padua, Italy; 3 Institute of Radiology, Department of Medicine, Padua University Hospital, University of Padua, Padua, Italy; 4 Department of Biology, University of Padua, Padua, Italy

**Keywords:** Hepatocellular carcinoma, Sarcopenia, Cirrhosis, Psoas muscle index, Textbook outcome, Nomogram

## Abstract

Sarcopenia, serving as a surrogate for frailty, is clinically significant in liver resection (LR) for elderly hepatocellular carcinoma (HCC) patients. Our study aims to assess sarcopenia’s impact, measured by Psoas Muscle Index (PMI), on postoperative outcomes. We retrospectively studied patients aged ≥ 60 years who underwent LR for HCC between 2014 and 2018. PMI, derived from preoperative CT scans, and Textbook Outcome (TO) for LR were assessed. A nomogram predicting overall survival (OS) was developed via multivariable analysis. Of the 149 eligible HCC patients, the median PMI was 7.225 cm^2^/m^2^ in males and 4.882 cm^2^/m^2^ in females, with 37 (24.8%) patients identified as sarcopenic. Mortality was significantly associated with sarcopenia (HR 2.15; *p* = 0.032), MELD ≥ 10 (HR 3.13; *p* = 0.001), > 3 HCC nodules (HR 4.97; *p* = 0.001), and Clavien–Dindo ≥ 3 complications (HR 3.38; *p* < 0.001). Sarcopenic patients had a 5-year OS of 38.8% compared to 61% for non-sarcopenic individuals (*p* = 0.085). Achieving TO correlated with higher OS (*p* = 0.01). In sarcopenic cases, the absence of postoperative complications emerged as a limiting factor. Sarcopenic patients failing to achieve TO had worse OS compared to non-sarcopenic and TO-achieving counterparts (5-year OS 18.5%; *p* = 0.00039). Sarcopenia emerges as a prognostic factor for LR outcomes in elderly HCC patients. Postoperative complications in sarcopenic patients may compromise oncological outcomes.

## Introduction

The elderly population has steadily increased due to advancements in medicine with over 50% of primary and metastatic liver cancers occurring in individuals aged over 65 [1]. Liver resection (LR) and liver transplantation (LT) are the only potential curative treatments for hepatocellular carcinoma (HCC), and with surgical advancements, hepatic resection for elderly HCC patients has become safer.

Respiratory complications [2], cardiac events [3], delirium [2–4], and discharge to rehabilitation facilities [4] pose significant challenges for hepatic resection in the elderly. Additionally, HCC patients often have underlying cirrhosis that worsens their clinical status and anticipates their biological aging process [5]. Therefore, preoperative risk assessment is crucial for elderly patients undergoing LR for HCC. Frailty, defined as “a biological syndrome of decreased reserve and resistance to stressors,” has been recognized in the geriatric population and is pertinent to older surgical patients due to the stress surgery imposes on physiological homeostasis [6–8]. The preoperative assessment of frailty includes clinical and radiological tests evaluating the patient’s overall status. Tests such as the Mini-Mental State Examination (MMSE) assess cognitive function, while the Short Physical Performance Battery (SPPB) and skeletal muscle area [9–12] assess strength, resistance, and muscle atrophy (sarcopenia). Sarcopenia, easily assessed through a pre-operative CT-scan measuring the skeletal muscle area, has been established as a surrogate for frailty [9, 13–15]. Notably, in the context of HCC patients, both aging and liver disease contribute to the development of sarcopenia [16, 17]. Furthermore, HCC, arising in cirrhotic livers, necessitates treatment decisions based on tumor burden and underlying disease grade in the perspective of the best survival benefit achievable [18–20].

This study aims to explore whether sarcopenia, estimated by the Psoas Muscle Index (PMI), rather than age, impacts postoperative outcomes after hepatic resection for HCC in elderly patients.

## Methods

This retrospective cohort study utilized a prospectively updated database of LR performed for HCC at a single institution. Eligible patients were aged ≥ 60 years with an elective indication for LR for HCC between January 2014 and December 2018. The timeframe was selected based on the availability of radiological computed tomography (CT) images on picture archiving and communication systems (PACS): no radiological images were stored before 2014.

**Fig. 1:**
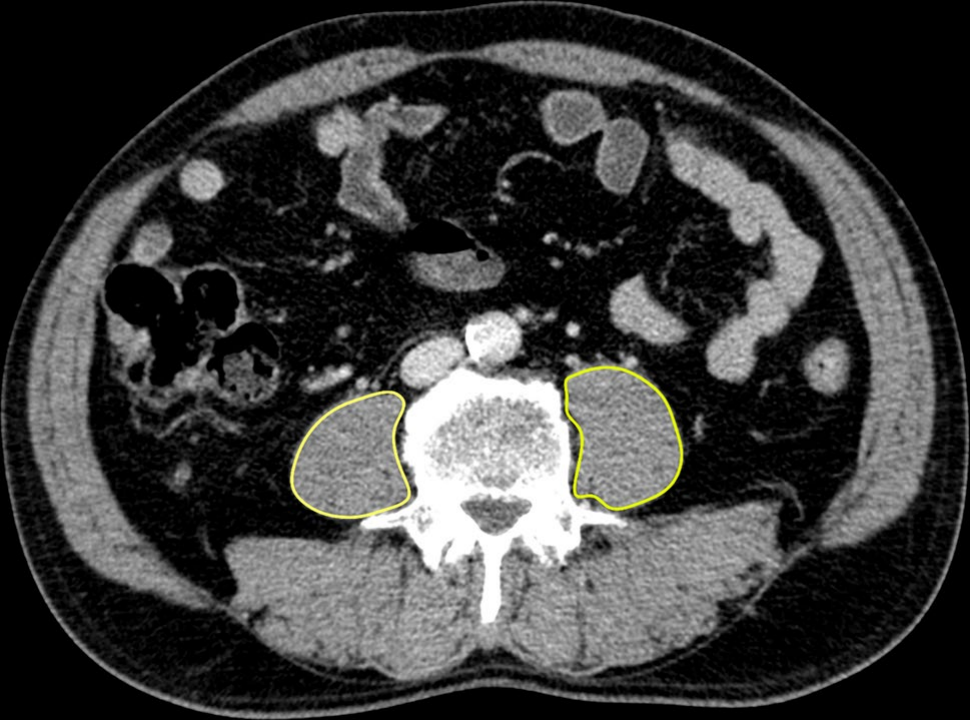
Psoas muscle area (PMA) measurement on a preoperative CT scan cross-section, calculated at the level of the 3rd lumbar vertebra. Psoas muscle margins are manually delineated to obtain both surface areas (yellow lines)

The study population was chosen so that both age-related and disease-related components of sarcopenia are captured (age-related sarcopenia is absent in younger adults) limiting the bias related to uncontrollable biological factors [5, 17]. To further minimize biases and confounding factors related to sarcopenia, exclusion criteria included the availability of inadequate radiological images, a timeframe between the last CT-scan and LR > 3 months, and previous LR for HCC. To assess muscle mass, all CT-scans were reviewed by a single expert radiologist blinded of the clinical patients’ data: a cross-section was selected at the level of the third or fourth lumbar vertebra and a manually traced Region of Interest (ROI) of the two psoas muscles was used to obtain both areas, by using an open-source medical image viewer (Horos) (Fig. 1). The Psoas Muscle Area (PMA) was then calculated as the sum of the areas, normalized for patients’ height (PMA/m^2^) to generate the Psoas Muscle Index (PMI). PMA and PMI values were stratified by sex and sarcopenia was censored at the 25th percentile of the distribution of PMI for women and men.

Demographic characteristics, preoperative laboratory values, operative and post-operative variables were collected.

Demographic characteristics of the population comprise age, gender, body mass index (BMI), American Society of Anesthesiology (ASA) grade, presence of cirrhosis, hepatitis C virus (HCV) infection status, history of previous surgery and history of previous chemotherapy with tyrosine-kinase inhibitors (TKI). An age cut-off value of 70 years was used to identify chronologically elderly patients. Therefore, age was managed and analyzed as a dichotomous variable. The preoperative laboratory comprises platelets count and alpha-fetoprotein (AFP) value. Operative and postoperative variables collected for the study were: surgical approach (open, laparoscopic), LR type (major resection involves three or more Couinaud’s segments [21, 22]), operative time, intraoperative blood loss, intraoperative blood transfusion, intraoperative incidents according to Oslo criteria [23], margin status, number of nodules and diameter of the major nodule, intensive care unit (ICU) stay, length of hospital stay (LOS), postoperative complications (classified according to Clavien–Dindo [24]), 30-days readmission and disease recurrence. Recurrence has been censored at its first appearance in any site at CT-scan during follow-up.

Furthermore, the Textbook Outcome (TO) for LR was calculated for each patient based on 6 operative items: absence of intraoperative incidents grade ≥ 2 according to Oslo criteria [23], no bile leak grade ≥ B according to the severity grading of the International Study Group of Liver Surgery [25], no complications grade ≥ 3 according to Clavien–Dindo, no readmission ≤ 30 days after discharge, no in-hospital mortality, and R0 resection margin, as previously described by Görgec et al. [26]. Overall survival (OS) was calculated from the time of surgery to the last follow-up date or death, HCC-related death was censored if the patient died due to tumor recurrence/progression, and disease-free survival (DFS) from the time of surgery to recurrence. Finally, a nomogram to predict OS probability was implemented based on the results of the multivariable analysis.

The study was conducted in accordance with the ethical guidelines of the 2013 revised Declaration of Helsinki. The study was approved by the ethics board of Territorial Ethics Committee Central—Eastern Veneto Area (CET—ACEV)—Regional Resolution No. 330/2023 (protocol number 448n/A0/23, 18 January 2024); each patient gave written consent for every procedure performed in the hospital, and for the use of data for research and publication purposes, and all procedures were performed in accordance with the Declaration of Istanbul. No one received compensation or was offered any incentive for participating in this study.

### Statistical analysis

Values for categorical variables were expressed as totals and percentages whereas for continuous variables they were expressed as medians and interquartile ranges (IQR). Statistical analyses were performed using Pearson’s chi-squared test or Fisher’s test for categorical variables and the Wilcoxon rank sum test for continuous variables.

The length of follow-up was calculated from the date of surgery to the date of patient death or the latest follow-up date for those still alive (overall survival—OS). The duration of follow-up and survival was expressed as median (IQR). Survival and recurrence curves were calculated using the Kaplan–Meier technique and compared with the log-rank test.

The univariable and multivariable Cox proportional-hazard models were used to evaluate variables associated with OS and DFS.

A *p*-value < 0.05 was considered to indicate statistical significance; variables with a *p*-value < 0.1 were considered of marginal statistical significance. Statistical analyses were performed using R, RStudio 4.3 (2023).

## Results

Between January 2014 and December 2018, 307 patients aged ≥ 60 years old underwent LR for HCC at the General Surgery 2 Hepato-pancreato-biliary Surgery and Liver Transplantation of Padua University Hospital, Padua, Italy.

Based on the selected exclusion criteria, we excluded 100 patients for inadequate radiological images (the only MRI was available, or only CT-scan of the upper abdomen); 53 patients for a timeframe > 3 months between the last CT-scan and surgery; 5 patients due to a previous LR for HCC. One-hundred-and-forty-nine patients were eligible for the study.

One-hundred- and-twelve (75%) patients were male, median age was 70 (65.6–75.8), median BMI was 26 (23.6–29.4) Kg/m^2^, 117 (78.5%) had a diagnosis of cirrhosis, 19/117 (16.2%) scored Child–Pugh class B, and 54 (36%) patients were hepatitis C virus (HCV) positive. Median PMA was 21.23 (18.01–23.65) cm^2^ in male patients and 12.95 (11.66–15.50) cm^2^ in females, while median PMI was 7.225 (6.282–8.140) cm^2^/m^2^ in male patients and 4.882 (4.413–5.720) cm^2^/m^2^ in female. In the male population, the 25th percentile was censored at 6.282 cm^2^/m^2^, while in the female population the 25th percentile of PMI was 4.413 cm^2^/m^2^. According to those cut-off values, 28 (25%) males and 9 (24.3%) females were deemed sarcopenic. Overall, 37 (24.8%) patients were considered sarcopenic. A BMI ≥ 30 kg/m^2^ was registered only in 3 (8.1%) out of 37 sarcopenic patients so no analysis of sarcopenic obesity was attempted.

Sarcopenic patients were older (72.0 [67.9, 77.3]; *p* = 0.027), had a lower BMI (25.1 [21.3,27.1]; *p* = 0.026), and a greater ASA score (≥ 3, 25/34 [74%]; *p* = 0.043) compared to non-sarcopenic. No differences were observed in terms of the patient's medical history, tumor, and intraoperative characteristics. Even though sarcopenic patients had longer ICU-stay (> 1 day, 16/37 [43%]; *p* = 0.006), no differences were observed in postoperative outcomes compared to non-sarcopenic. Table 1 resumes the main perioperative and postoperative characteristics of the two cohorts.

**Table 1:** Demographical, perioperative and postoperative characteristics

Characteristic	Non sarcopenic *N* = 112^*1*^	Sarcopenic *N* = 37^*1*^	*p*^2^
Age ≥ 70 years	50/112 (45%)	24/37 (65%)	0.033
Gender (male)	84/112 (75%)	28/37 (76%)	0.93
BMI (Kg/m^2^) (*missing)	26.7 (24.2, 29.8) *23	25.1 (21.3, 27.1) *6	0.026
ASA ≥ 3 (*missing)	56/104 (54%) *8	25/34 (74%) *3	0.043
Cirrhosis	89/112 (79.5%)	28/37 (76%)	0.71
Child–Pugh grade B	15/112 (13.4%)	4/37 (11%)	0.78
MELD ≥ 10 (*missing)	25/111 (23%) *1	4/37 (11%)	0.12
HBV positive	12/112 (11%)	8/37 (22%)	0.10
HCV positive	39/112 (35%)	15/37 (41%)	0.53
AFP (ng/L) (*missing)	10.9 (4.7, 88.4) *9	44.5 (6.0, 512.0) *4	0.060
Platelet ×10^9^/L (*missing)	148.0 (97.0, 206.0) *1	158.0 (112.0, 256.0)	0.11
Platelet < 100 ×10^9^/L	30/111 (27%)	6/37 (16%)	0.18
History of TKI therapy	8/112 (7.1%)	2/37 (5.4%)	> 0.99
History of laparoscopic surgery	9/112 (8.0%)	1/37 (2.7%)	0.45
History of open surgery	5/112 (4.5%)	3/37 (8.1%)	0.41
History of hepatic MWA	8/112 (7.1%)	1/37 (2.7%)	0.45
Diameter of the largest nodule			
≤ 2 cm	11/112 (9.8%)	6/37 (16%)	0.17
> 2 and ≤ 5 cm	52/112 (46%)	11/37 (30%)	
> 5 cm	49/112 (44%)	20/37 (54%)	
Numbers of nodules > 3	8/112 (7.1%)	2/37 (5.4%)	> 0.99
Milan out	58/112 (52%)	22/37 (59%)	0.42
Intraoperative characteristics			
MILS approach	55/112 (49%)	18/37 (49%)	0.96
Major hepatectomy	29/112 (26%)	11/37 (30%)	0.65
Concomitant MWA	43/112 (38%)	9/37 (24%)	0.12
Pringle manouvre (yes)	18/112 (16%)	6/37 (16%)	0.98
Blood loss (mL) (*missing)	300.0 (100.0, 600.0) *40	200.0 (25.0, 850.0) *14	0.85
Operative time (min) (*missing)	237.5 (176.2, 340.0) *2	250.0 (190.0, 375.0)	0.31
Postoperative characteristics			
Resection margin R1	28/112 (25%)	6/37 (16%)	0.27
ICU-stay > 1 day	23/112 (21%)	16/37 (43%)	0.006
LOS (days)	7.0 (5.0,10.0)	8.0 (5.0,12.0)	0.23
Post-operative complications (yes)	68/112 (61%)	26/37 (70%)	0.30
Clavien–Dindo ≥ 3	17/112 (15%)	7/37(19%)	0.59
Bile leak (yes)	2/112 (1.8%)	2/37 (5.4%)	0.26
Readmission within 30 POD	6/112 (5.4%)	3/37 (8.1%)	0.69
Mortality within 30 POD	2/112 (1.8%)	3/37 (8.1%)	0.10
In-hospital mortality or within 90 POD (*missing)	6/111 (5.4%) *1	6/36 (17%) *1	0.072
HCC recurrence	81/112 (72%)	22/37 (59%)	0.14

*BMI* body mass index, *ASA* American Society of Anesthesiology, *MELD* model for end-stage liver disease, *HBV* hepatitis B virus, *HCV* hepatitis C virus, *AFP* alpha-fetoprotein, *TKI* tyrosine-kinase inhibitors, *MWA* microwave ablation, *MILS* minimally invasive liver surgery, *ICU* intensive care unit, *LOS* length of hospital stay, *POD* postoperative day, *HCC* hepatocellular carcinoma

^1^Median (IQR); n/N (%), ^2^Wilcoxon rank sum test; Pearson's Chi-squared test; Fisher's exact test

After uni- and multi-variable analysis, only BMI (OR 0.89; 95% CI 0.79, 0.98; *p* = 0.027) and ASA grade ≥ 3 (OR 3.33; 95% CI 1.22, 10.3; *p* = 0.026) were statistically significant associated with the presence of sarcopenia in the whole population (Table 2).

**Table 2:** Uni- and multi-variable analysis of characteristics associated to the presence of sarcopenia

Characteristic	Univariable			Multivariable		
	OR^*1*^	95% CI^1^	*p*	OR^1^	95% CI^1^	*p*
Age ≥ 70 years	2.29	1.07, 5.06	0.035	2.36	0.92, 6.34	0.078
BMI (Kg/m^2^)	0.88	0.79, 0.97	0.015	0.89	0.79, 0.98	0.027
Gender (male)	1.04	0.45, 2.57	> 0.9			
ASA ≥ 3	2.38	1.04, 5.85	0.046	3.33	1.22, 10.3	0.026
Cirrhosis	0.85	0.36, 2.12	0.7			
Child–Pugh grade B	0.77	0.21, 2.32	0.7			
MELD ≥ 10	0.42	0.12, 1.18	0.13			
HBV positive	2.30	0.83, 6.11	0.10			
HCV positive	1.28	0.59, 2.73	0.5			
AFP (ng/L)	1.00	1.00, 1.00	0.11			
Platelet ×10^9^/L	1.00	1.00, 1.01	0.063	1.00	1.00, 1.01	0.12
History of TKI therapy	0.74	0.11, 3.14	0.7			
History of laparoscopic surgery	0.32	0.02, 1.78	0.3			
History of open surgery	1.89	0.37, 8.11	0.4			
History of hepatic MWA	0.36	0.02, 2.07	0.3			
Diameter of the largest nodule						
≤ 2 cm	–	–				
> 2 and ≤ 5 cm	0.39	0.12, 1.32	0.12			
> 5 cm	0.75	0.25, 2.42	0.6			
Number of nodules > 3	0.74	0.11, 3.14	0.7			
Milan Out	1.37	0.65, 2.94	0.4			

*OR* odds ratio, *CI* Confidence Interval, *BMI* body mass index, *ASA* American Society of Anesthesiology, *MELD* model for end-stage liver disease, *HBV* hepatitis B virus, *HCV* hepatitis C virus, *AFP* alpha-fetoprotein, *TKI* tyrosine-kinase inhibitors, *MWA* microwave ablation

To investigate the risks factors associated with the probability of patients death, a Cox proportional-hazard regression analysis was performed, and the multivariable analysis showed a statistically significant association with sarcopenia (HR 2.15; 95% CI 1.07, 4.33; *p* = 0.032), MELD ≥ 10 (HR 3.13; 95% CI 1.55, 6.31; *p* = 0.001), HCC nodules > 3 (HR 4.97; 95% CI 1.86, 13.3; *p* = 0.001) and Clavien–Dindo ≥ 3 postoperative complication (HR 3.38; 95% CI 1.69, 6.75; *p* < 0.001) (Table 3).

**Table 3:** Uni- and multi-variable analysis of characteristics associated with the probability of death

Characteristic	Characteristics associated with the probability of patients’ death						Characteristics associated with the probability of HCC-related death					
	Univariable			Multivariable			Univariable			Multivariable		
	HR	95% CI	*p*	HR	95% CI	*p*	HR	95% CI	*p*	HR	95% CI	*p*
Age ≥ 70 years	1.17	0.65, 2.12	0.6	1.44	0.73, 2.85	0.3	0.92	0.45, 1.85	0.8	1.10	0.50, 2.43	0.8
BMI (Kg/m^2^)	0.94	0.87, 1.01	0.11				1.01	0.93, 1.10	0.8			
Gender (male)	0.92	0.46, 1.87	0.8				0.63	0.29, 1.38	0.2			
ASA ≥ 3	1.37	0.71, 2.66	0.3				1.14	0.52, 2.49	0.7			
Sarcopenic PMI (yes)	1.74	0.92, 3.28	0.089	2.15	1.07, 4.33	0.032	1.13	0.49, 2.62	0.8	1.44	0.58, 3.59	0.4
Cirrhosis	0.94	0.49, 1.81	0.9				0.65	0.32, 1.35	0.2			
Child–Pugh grade B	2.63	1.28, 5.37	0.008				1.76	0.66, 4.65	0.3			
MELD ≥ 10	2.46	1.28, 4.73	0.007	3.13	1.55, 6.31	0.001	1.82	0.78, 4.25	0.2	1.99	0.81, 4.88	0.13
HBV positive	1.06	0.42, 2.69	> 0.9				0.59	0.14, 2.46	0.5			
HCV positive	1.24	0.67, 2.29	0.5				0.91	0.42, 1.98	0.8			
AFP (ng/L)	1.00	1.00, 1.00	0.11				1.00	1.00, 1.00	> 0.9			
Platelet ×10^9^/L	1.00	1.00, 1.00	0.8				1.00	1.00, 1.00	0.3			
History of TKI therapy	0.98	0.23, 4.09	> 0.9				1.59	0.37, 6.80	0.5			
History of laparoscopic surgery	0.97	0.23, 4.03	> 0.9				0.73	0.10, 5.40	0.8			
History of open surgery	1.27	0.39, 4.13	0.7				1.93	0.58, 6.43	0.3			
History of hepatic MWA	1.30	0.31, 5.44	0.7				1.03	0.14, 7.71	> 0.9			
Diameter of the largest nodule												
≤ 2 cm	–	–					–	–				
> 2 and ≤ 5 cm	1.27	0.47, 3.46	0.6				1.72	0.49, 6.07	0.4			
> 5 cm	1.78	0.67, 4.77	0.2				2.24	0.64, 7.84	0.2			
Number of nodules > 3	3.35	1.29, 8.74	0.013	4.97	1.86, 13.3	0.001	4.69	1.57, 14.0	0.006	5.91	1.90, 18.4	0.002
Milan Out	1.59	0.87, 2.91	0.13				1.61	0.78, 3.29	0.2			
Open surgical approach	1.40	0.76, 2.58	0.3				1.18	0.58, 2.42	0.6			
Major hepatectomy	1.38	0.73, 2.61	0.3				1.90	0.92, 3.91	0.083			
Concomitant MWA	1.29	0.70, 2.38	0.4				1.16	0.55, 2.43	0.7			
Pringle manouvre (yes)	1.01	0.45, 2.26	> 0.9				1.04	0.40, 2.71	> 0.9			
Blood loss (mL)	1.00	1.00, 1.00	0.5				1.00	1.00, 1.00	0.3			
Operative time (min)	1.00	1.00, 1.00	0.7				1.00	1.00, 1.00	0.2			
Resection margin R1	1.55	0.78, 3.09	0.2				1.44	0.61, 3.39	0.4			
ICU-stay > 1 day	1.87	0.98, 3.58	0.059				2.06	0.95, 4.49	0.068			
LOS (days)	1.04	1.02, 1.06	< 0.001				1.04	1.01, 1.06	0.013			
Post-operative complications (yes)	1.46	0.78, 2.72	0.2				1.44	0.69, 3.02	0.3			
Clavien-Dindo ≥ 3	2.79	1.43, 5.44	0.003	3.38	1.69, 6.75	< 0.001	1.83	0.75, 4.49	0.2	2.22	0.87, 5.62	0.094
Bile leak (yes)	1.33	0.32, 5.51	0.7				1.88	0.44, 7.93	0.4			
Readmission within 30 POD	1.65	0.58, 4.68	0.3				0.53	0.07, 3.95	0.5			

*HR* hazard ratio, *CI* confidence interval, *BMI* body mass index, *ASA* American Society of Anesthesiology, *PMA* psoas muscle area, *PMI* psoas muscle index, *MELD* model for end-stage liver disease, *HBV* hepatitis B virus, *HCV* hepatitis C virus, *AFP* alpha-fetoprotein, *TKI* tyrosine-kinase inhibitors, *MWA* microwave ablation, *MILS* minimally invasive liver surgery, *ICU* intensive care unit, *LOS* length of hospital stay, *POD* postoperative day

In the current setting of aged (≥ 60 years) patients, given the non-negligible probability of death for causes other than HCC (e.g.: cerebrovascular or cardiovascular disease), further Cox Proportional-Hazard regression analysis was performed to investigate the HCC-related death. This latter multivariable analysis showed that the only factor associated with HCC-related death was HCC nodules > 3 (HR 5.91; 95% CI 1.90, 18.4; *p* = 0.002). (Table 3). Furthermore, history of TKI therapy (HR 3.26; 95% CI 1.59, 6.67; *p* = 0.001), HCC nodules > 3 (HR 2.74; 95% CI 1.34, 5.61; *p* = 0.006), and positive resection margin (R1) (HR 1.70; 95% CI 1.07, 2.68; *p* = 0.023) were independent risk factors for reduced DFS at Cox proportional-hazard regression analysis.

**Fig. 2:**
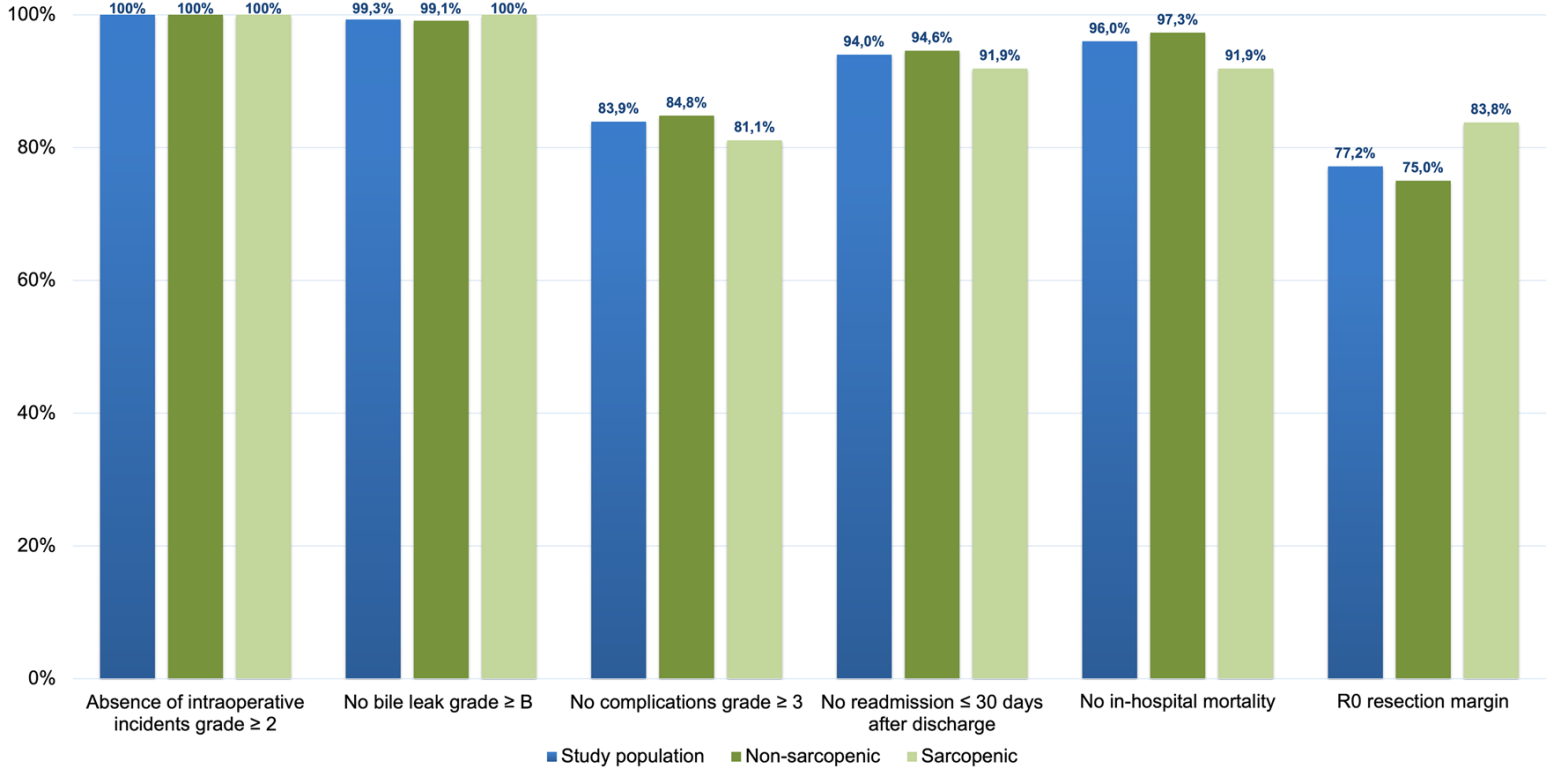
The proportion of patients who achieved each desired health outcome forming the textbook outcome (TO) in the whole population, and the two groups individually

In our study population, TO was achieved in 98 (66%) patients. Twenty-five out of 37 (68%) sarcopenic patients achieved TO, and 73 out of 112 (65%) non-sarcopenic achieved TO (*p* = 0.79). The achievement of each TO item was calculated separately with cumulative percentages to identify which indicator was the main limiting factor for accomplishing TO. The R0 resection margin is the item found to have the lowest achievement incidence compared to the others (77.2%). Interestingly, in sarcopenic patients, the main limiting factor is the absence of postoperative complications grade ≥ 3 (81.1%) (Fig. 2).

### Survival analysis

After LR, the 1-, 3- and 5-year overall survival (OS) of our aged HCC patient population were 85.9%, 65.9%, and 56.3% respectively with a median follow-up of 16.7 (IQR 6.1; 37.1) months.

**Fig. 3:**
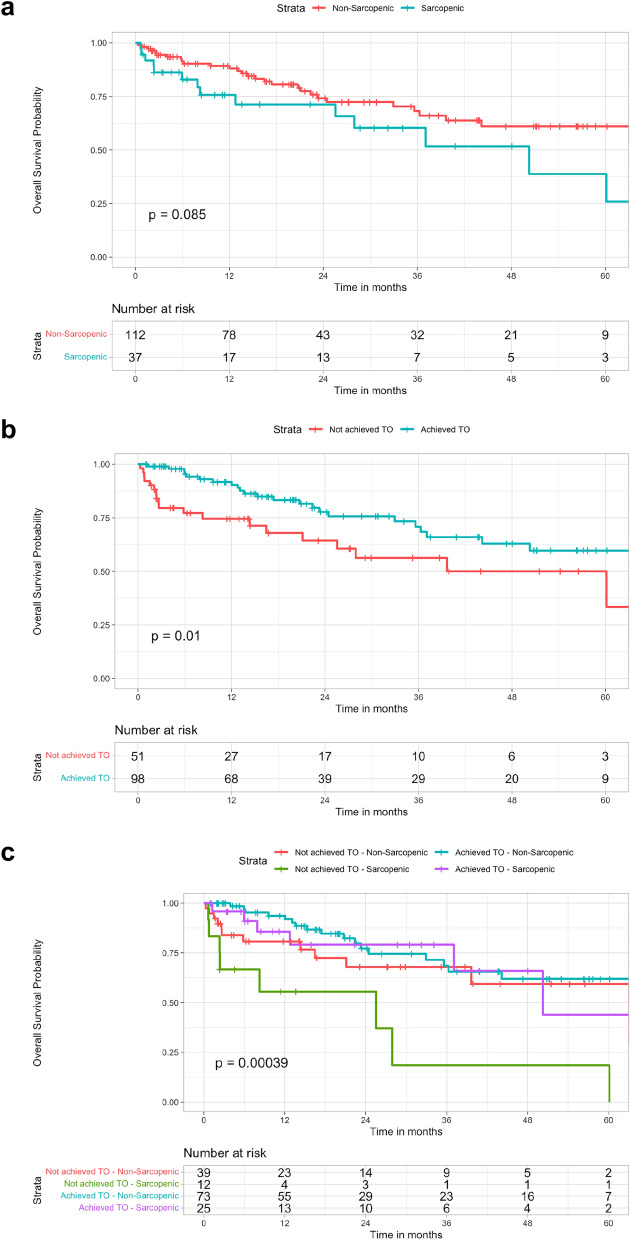
Kaplan–Meier survival curves of the study population stratified according to sarcopenia status (**a**); Kaplan–Meier survival curves of the study population stratified according to textbook outcome (TO) achievement (**b**); Kaplan–Meier survival curves of the study population stratified according to both sarcopenia status and TO achievement (**c**)

Sarcopenic patients had an OS of 75.7%, 60.3%, and 38.8% at 1, 3, and 5 years, respectively, compared to 89.2%, 68.2% and 61% at 1, 3, and 5 years respectively registered in non-sarcopenic (*p* = 0.085) (Fig. 3a).

Patients who reached TO showed a significantly higher OS compared to patients who didn’t (TO Achieved patients 1-, 3-, and 5-year OS was 91.7%, 70.9%, and 59.7%, respectively; TO Not-achieved patients 1-, 3-, and 5-year OS was 74.6%, 56.3%, and 50%; *p* = 0.01) (Fig. 3b). Interestingly, when TO achievement was stratified according to sarcopenia status, sarcopenic patients who did not achieve TO had worse survival outcomes compared to both non-sarcopenic (regardless of TO status) and sarcopenic who achieved TO (OS at 1, 3, and 5 years 55.6%, 18.5% and 18.5% respectively; *p* = 0.00039) (Fig. 3c).

### Nomogram

**Fig. 4:**
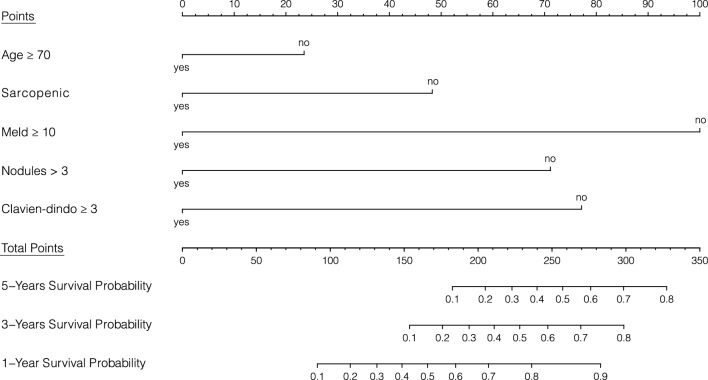
Nomogram to predict post-operative survival probability

We developed a nomogram to predict post-operative survival probability based on the multivariable Cox proportional-hazard regression analysis. Hence, age ≥ 70, sarcopenia, MELD ≥ 10, HCC nodules > 3, and Clavien–Dindo ≥ 3 postoperative complications were the selected items, with each variable being assigned a score on a point scale. By adding the scores and by locating the total score on the total points scale, a straight line could be drawn perpendicularly downwards to identify the probabilities of 1-, 3-, and 5-year OS (Fig. 4). These predictive probabilities could be used to predict the outcome of LR for HCC for an individual patient.

## Discussion

In an aging oncological population, surgical treatments for hepatocellular carcinoma (HCC) are increasingly offered to elderly patients. Aged HCC patients suffer from a condition of frailty that depends not only on their age per se but also on tumor, cirrhosis, and muscle atrophy; conditions that are often interconnected. Indeed, excluding patients from potentially curative treatments only for their age is often unjustifiable, given the good results of LR in the elderly [27–29].

Frailty is a multiparametric definition that relies both on functional status and muscle atrophy, which was already linked to poor treatment outcomes. However, assessing patients’ frailty is demanding. Sarcopenia can be easily assessed through the Psoas Muscle Index (PMI), emerged as a surrogate for frailty further confirming the increased role of medical radiology in improving patients’ management [29, 30]. Despite various definitions of sarcopenia, PMI stands out as a surgeon-friendly tool, aiding in preoperative assessment, considering the real-life experience of outpatient visits and preoperative assessment in a high-volume hepatobiliary center. In our study population, male patients with PMI < 6.282 cm^2^/m^2^ and female patients with PMI < 4.413 cm^2^/m^2^ were deemed sarcopenic. Interestingly, age was not associated with the presence of sarcopenia, but BMI and ASA grade ≥ 3 did, reinforcing the concept that sarcopenia works as a strong surrogate of patients' pathophysiological condition, regardless of the age.

Moreover, patient survival after LR is independent of age. Our study showed that sarcopenia, MELD, number of nodules, and postoperative complications were the variables significantly associated with OS. Notably, when competing-risk analysis of OS for HCC-related death was performed, only tumor burden (number of nodules > 3) is an independent factor; this result further strengthens previous findings that patient prognosis is mostly driven by HCC recurrence and that sarcopenic patient is more exposed to critical events that can lead to death independently from HCC. Moreover, sarcopenia was not associated with HCC recurrence; not surprisingly, history of TKI therapy, HCC nodules > 3, and R1 resection were independent risk factors for reduced DFS.

In the specific field of HCC on cirrhosis, there is still little evidence on the usefulness of prehabilitation programs for patients before liver resection. Still, published studies have shown that interventions to reduce or mitigate preoperative frailty in these patients improve disease-free and long-term survival [31, 32]. Furthermore, patient performance during prehabilitation correlates with the outcome of liver resection [31–33]. From this perspective, the results of our study confirm the importance of prehabilitation programs and should stimulate the design of targeted prospective studies.

Unfortunately, many sarcopenia definitions have been developed and there is yet no agreement on which is the best predictor and which cut-off value to use [9]. In the current setting, it is very difficult to achieve a unique result because the authors investigated sarcopenia relative to the surgical procedure and not as a patient condition per se so that in literature are reported different cut-off values for different HCC treatments, and this is partially justified by retrospective studies and need to compare homogeneous data [10–12]. In this regard, our study is not an exception, and this is its main limit, among the retrospective analysis and the limited sample volume. Nevertheless, our study has value in demonstrating the connection between sarcopenia and resection outcome from a new perspective. Sarcopenic patients who did not achieve TO had significantly poorer survival outcomes compared to other patients. Given that the absence of postoperative complication grade ≥ 3 is the main limiting factor for TO achievement in those patients, particular attention should be reserved for sarcopenic ones in terms of prevention of postoperative events, prompting refinements in preoperative assessment, surgical strategy, and therapeutical allocation based on sarcopenic status instead of chronological age.

## Conclusions

Sarcopenia, rather than age, predicts the outcome of LR for HCC in the elderly. Sarcopenia assessed through PMI, is a valuable predictor of outcomes in elderly patients undergoing LR for HCC. Incorporating sarcopenia into preoperative assessments allows for tailored surgical strategies, emphasizing the need for preventive measures to avoid complications and improve overall clinical outcomes.

## Data Availability

The datasets generated during and/or analyzed during the current study are not publicly available but are available from the corresponding author on reasonable request.
